# A Novel Medical Device for Airway Clearance

**DOI:** 10.3390/jcm14030907

**Published:** 2025-01-30

**Authors:** Nir Helper, Moshe Ashkenazi, Gil Sokol, Adi Dagan, Ori Efrati

**Affiliations:** 1 Pediatric Pulmonology and National CF Center, Edmond and Lily Safra Children’s Hospital, Sheba Medical Center, Tel-Hashomer, Ramat Gan 5262000, Israel; 2 Faculty of Medicine, Tel-Aviv University, Tel-Aviv 6997801, Israel

**Keywords:** cystic fibrosis, bronchiectasis, airway clearance, LibAirty, BiPAP

## Abstract

**Background**: Airway clearance techniques are a key element in the daily treatment of people with bronchiectasis. There are several methods and devices to assist in effective airway clearance. We investigated LibAirty, a novel medical device, and compared it with the common practice performed today. **Methods**: Twenty adults enrolled, and each one had three different treatments in a randomized order: a human respiratory physiotherapist, a High-Frequency Chest Wall Oscillator, and LibAirty with BiPAP. The outcome parameters were mucus weight and a questionnaire. Further studies were performed to investigate LibAirty with hypertonic saline (HS) inhalation and using the device as a standalone. **Results**: No adverse events were recorded. The sputum amount expectorated in all arms using LibAirty was 14.4 ± 11.1 g with BIPAP, 16.4 ± 7 g with HS, and 11.3 ± 4.1 g for the standalone treatment. For HFCWO, 4.45 ± 3.28 g was obtained, and for CPT, 15.9 ± 11.1 g was obtained. The amount obtained by LibAirty (all arms) was significantly higher than HFCWO. **Conclusions**: All arms of LibAirty were superior to HFCWO and similar to the human physiotherapist. Further studies should be performed to investigate the long-term effects of LibAirty.

## 1. Introduction

Airway mucus obstruction is a key characteristic of chronic obstructive lung disease with various etiologies, from genetic disorders such as cystic fibrosis (CF) to other diseases that can result in bronchiectasis (non-CF bronchiectasis) [1,2]. The former is the most common lethal genetic disease in white populations, and the latter is the third most common chronic airway disease worldwide. Although the pathophysiology is different, the vicious infection cycle is similar; chronic infection damages mucociliary clearance, increasing the chances of further infection, deteriorated lung function, and even end-stage lung disease. An alternative way to monitor CF lung disease was described elsewhere [3]. Abramit et al. described the essential features of normal and pathologic mucus and the contribution of deferent mucins and mediators to airway clearance [4]. As the disease progresses, exacerbations (e.g., the worsening of clinical symptoms including decreased lung function and lower quality of life as well as an increase in mucus production and cough) become more frequent, leading to frequent hospital admissions [5] and an increased economic burden. According to a study by Weycker et al., in 2013, there were approximately 522,000 adults diagnosed with bronchiectasis in the US [6] with an estimated annual cost of USD 5000 in Europe and USD 35,000 in the US [7]. Correspondingly, according to the European and North American CF registries, there are approximately 80,000 patients with CF in these regions, with an estimated annual cost of USD 82,000 per patient.

Considering the high prevalence, its ramification on quality of life and significant financial burden on the health system, evidence-based interventions that reduce exacerbations and improve quality of life are necessary.

To date, the main goal for the management of bronchiectasis (PwBE), considering that a substantial sub-group of bronchiectasis patients are those with CF (PwCF), is to promote airway clearance via antibiotics, inhaled bronchodilators, corticosteroids, mucolytic agents, and the inhalation of hypertonic saline (HS) 3% [8,9,10]; furthermore, a key element of management includes a wide family of devices and breathing techniques known as airway clearance techniques (ACTs) [11]. Specifically, the goal of ACTs is to remove thick mucus from the airways, breaking the vicious cycle of infection and loss of function previously mentioned, preserving lung function from deterioration, and delaying disease progression [12].

A study by Munoz et al. [13] compared the effect of an ACT versus a placebo treatment in PwBE over the span of a year. The results showed that the ACT group had fewer exacerbations and improved quality of life compared to the placebo [13].

Similarly, Macllwaine et al. [14] studied the effect of Positive Expiratory Pressure (PEP) therapy compared to High-Frequency Chest Wall Oscillation (HFCWO) over the span of a year in PwCF, and results showed a relatively lower pulmonary exacerbation rate for the PEP therapy group and an increase in lung function for both treatments.

Interestingly, though, in recent years, the use of bBi-level positive airway pressure (BiPAP) ventilator devices adjunct to airway clearance in PwCF has been shown to improve the heterogeneity of air distribution in the lungs as well as the quality of life outcomes compared to other ACTs [15].

The BiPAP device prevents airway collapse during expiration, with the benefit of improving ventilation for the obstructed areas of the lungs via positive inspiratory pressure during the inspiratory phase of the breathing cycle [15,16,17].

Given the variety of airway clearance devices and breathing techniques, it is requisite to incorporate different breathing techniques in conjunction with different devices. A recent study by our group has shown a greater yield of mucus in PwCF using a mechanical insufflator exsufflator in conjunction with the autogenic drainage (AD) breathing technique compared to AD alone [18].

AD is a breathing technique used to clear secretions by adjusting the breathing volume, creating shearing forces that expel the mucus [16]; the application of AD can be performed autonomously or with the aid of a therapist (assisted autogenic drainage—AAD). AD has been shown to improve expectorated mucus and improve lung heterogeneity [2,16,19].

Although ACTs have been shown to improve mucus expectorated, lung function and quality of life reviews in recent years highlighted that the debate concerning the identification of the most effective ACT to treat people with mucus retention due to bronchiectasis or CF is still to be determined [19,20,21].

The LibAirty Airway Clearance System (Synchrony Medical Ltd., Or Yehuda, Israel) is a novel device designed to assist in the loosening and elimination of mucus from the airways. Inspired by the autogenic drainage airway clearance technique, in which adjusting the depth and location of lung volumes during respiration generates shearing forces induced by airflow, which loosen, mobilize, and move secretions from the peripheral towards the central airways, this system promotes breathing at different lung volumes. This is achieved by synchronizing sequential chest compressions delivered by an inflatable vest with controlled breathing, using a designated mobile application to control the treatment and provide guidance.

When we observe treatment from the point of view of the caregivers, we can see that respiratory therapist professionals report high rates of burnout, 79%, according to Miller et al. [22], from not working with adequate staffing [23]. As a consequence, between 2019 and 2020, the shortage of professionals increased to 31% [24], a process that was accelerated during the COVID-19 crisis, and, nowadays, estimations (ref D) [25] calculate that with the future increase in demand for respiratory therapists, the annual demand could reach 9400 professionals.

Therefore, we conducted a study to investigate the acute effect of three ACT modalities on PwBE (either CF or non-CF), comparing an HFCWO device and the incorporation of the AD technique with a BiPAP device assisted by an expert respiratory physiotherapist (PT) guiding the patients’ breathing pattern similarly to the AD technique with the BiPAP device using a novel device, e.g., the LibAirty Airway Clearance System. The aim of the study was to explore the efficacy of LibAirty compared to the common practice today.

## 2. Materials and Methods

### 2.1. Participants

Twenty adults treated at the pulmonary unit, Edmond and Lily Safra Children’s Hospital (Ramat Gan, Israel), were enrolled in the study.

The Inclusion criteria were adult patients older than 18 years of age with a confirmed diagnosis of cystic fibrosis or non-CF bronchiectasis who were treated regularly with ACT and clinically stable according to the physician’s assessment.

Exclusion criteria were forced expiratory volume for 1 s (FEV_1_) < 25% predicted, an inability to perform independent chest physiotherapy, history of Hemoptysis, acute exacerbation, known diagnosis of portal hypertension, Thrombocytopenia (<50,000), Coagulopathy (primary or secondary), any cardiac disease, severe osteoporosis (lower than -2SD), or rib fracture in the last 6 months.

### 2.2. Study Design

This prospective two-phase crossover study was approved by the Sheba Medical Center IRB (7096-20-SMC). All participants gave informed consent prior to conducting the initial measurements.

#### 2.2.1. Phase One

Participants were asked to arrive at the pulmonary unit for three different visits; each visit was composed of baseline testing (Pre), a twenty-minute airway clearance therapy session followed by repeated measures of the initial tests (Post). All pulmonary function tests were conducted by a professional technician blinded to the treatments. Furthermore, all treatments were supervised by an experienced physical therapist, and airway clearance treatments were assigned randomly using the “Research Randomizer” software (1st version).

Pre- and post-testing included spirometry, lung volumes, and surface oxygen saturation measured using a finger pulse oximeter (Nonin PalmSAT 2500, Plymouth, MN, USA). Additionally, the mucus expectorated during the treatment was collected and measured in grams. Moreover, participants were asked to evaluate their level of dyspnea and fatigue induced by the treatment on a ten-point rating scale [26,27]. Immediately after the treatment, participants were asked to evaluate their ease of breathing and assess the amount of mucus expectorated compared to their usual airway clearance therapy.

#### 2.2.2. Phase Two

Further investigation was conducted in order to better explore the airway clearance properties of the LibAirty airway clearance system. Two additional therapy sessions were added; the first included the LibAirty airway clearance system with nebulized HS 3%, and the second therapy with the LibAirty airway clearance system was used as a standalone therapy.

### 2.3. Airway Clearance Protocol

#### 2.3.1. Phase One

PT:

The PT treatments were performed utilizing the BiPAP device (VIVO 40 Breas, Stockholm, Sweden) via a face mask, set to an inspiratory positive airway pressure of 25 CM H_2_O and a positive end-expiratory pressure of 5 CM H_2_O. Participants were seated in the upright position while the physiotherapist stood behind them, placing their thumb on the clavicle with the palm facing the anterior axillary line.

The physiotherapist restricted the inflation of the chest wall, helping the participant maintain lower lung volumes; meanwhile, participants were instructed to breathe according to the AD breathing pattern.

Treatment started with 1–2 deep inhalations through the nose. Then, participants were instructed to exhale as much as possible and breathe in small volumes. When expectorations were heard or felt, the subject was guided to inhale larger volumes and cough. Expectorations were collected in a sterile cup and measured at the end of the treatment. Approximately 12–15 cycles were completed during each twenty-minute treatment session.

LibAirty with BiPAP:

The LibAirty™ Airway Clearance System vest was fitted to the participant, and a BiPAP was set to the same inspiratory and expiratory levels as the PT treatment. Participants were seated in the upright position and were provided with breathing instructions via a mobile application while the LibAirty vest’s alternating compressions restricted the chest wall. The pressure settings were adjusted to the individual’s tolerance, achieving a comfortable snug fit. The participants were instructed to cough using the system during the twenty-minute treatment. Approximately 12–15 cycles were completed during each twenty-minute treatment session.

HFCWO:

The HFCWO treatment session was provided with The Vest Airway Clearance System (Hillrom, Chicago, IL, USA), consisting of an inflatable vest. Participants were seated in the upright position with the vest fitted and the device set to an oscillating frequency of 13–15 HZ. Participants were instructed to breathe calmly with an open glottis.

The twenty-minute treatments were composed of four cycles of five minutes with the vest activated, followed by a one-minute pause; participants were encouraged to huff and cough during this time.

#### 2.3.2. Phase Two

LibAirty with nebulized HS:

Treatment included the LibAirty™ airway clearance system as described in phase one, while participants used a standard hospital nebulizer with 4 mL HS 3% instead of a BiPAP device. Approximately 12–15 cycles were completed during each twenty-minute treatment session.

LibAirty standalone:

This treatment included the LibAirty™ airway clearance system, as described in phase one, without any accompanying devices. Approximately 12–15 cycles were completed during each twenty-minute treatment session.

### 2.4. Outcome Measures

Mucus:

Participants were given a pre-weight specimen container and were asked to expectorate during the treatment into the container and at the end of the treatment, participants were asked to seal the container, then sputum was measured for weight in grams.

Lung function:

As a safety measure, lung function tests were performed before and after each treatment of phase one. Flow/volume maneuver (forced spirometry) was analyzed according to established guidelines inside the whole-body plethysmography (Carefusion, Hochberg. Germany, Masterscreen) [28]. We monitored forced expiratory volume in 1 s (FEV1), forced vital capacity (FVC), and forced expiratory flow between 25% and 75% of FVC (FEF25–75%).

Lung volume measurements performed as part of the whole-body plethysmography included total lung capacity (TLC); residual volume (RV); functional residual capacity (FRC); and the RV/TLC ratio. [29]

Fatigue:

The Borg dyspnea scale uses participants’ reports from 0 to 10 to rate dyspnea, where “0” means no symptoms and “10” represents maximum symptoms. [26,27] Similarly, the participants were asked to rate their level of fatigue caused by the treatment on a 0–10 scale, where “0” indicated no fatigue and “10” indicated exhaustion.

Dyspnea:

The rating of perceived exertion was assessed using a modified Borg scale in which “0” represents no effort and “10” represents maximal effort

### 2.5. Statistics

In order to characterize the cohort, descriptive statistics were performed for anthropometric data and lung function using the median and range. A paired *t*-test or model was used for the analyses of the different treatments and time points (pre–post) for variables such as lung function, the mucus expectorated, fatigue, and dyspnea.

Data were analyzed using SPSS (Version 28) software, and *p* < 0.05 was considered significant.

This is a pilot investigation trial. No statistical analysis was performed in the generation of the sample size; however, similar studies for these types of devices were reviewed to determine an adequate sample size of 17 [13].

## 3. Results

Twenty participants (eight males, 40%) enrolled in the study and completed phase one; eight were diagnosed with CF (40%); and twelve (60%) were diagnosed with non-CF bronchiectasis. Out of the twenty who completed phase one, twelve people (two PwCF and ten non-CF) also completed phase two.

The average age of the participants was 44 ± 21 years, and the average BMI was 22.4 ± 3.72 kg/m^2^. Among the participants, six had mild lung disease (median 94.5%, range 81–118% of predicated), eleven had moderate lung disease (median—58%, range 51–78% of predicted), and three subjects had severe lung disease (median 36%, range 28–33% of predicted). No adverse events occurred during the treatments. Baseline characteristics were recorded during the first visit, including anthropometric variables, lung function, and oxygen saturation at rest, as presented in Table 1.

**Figure 1:**
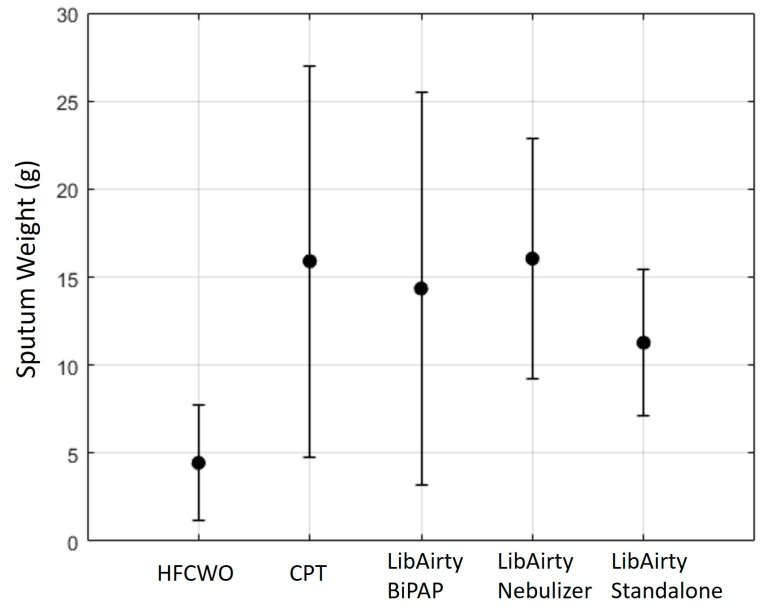
Sputum weight per session. HFCWO at 4.45 ± 3.28 g, CPT at 15.9 ± 11.1 g, LibAirty with BiPAP at 14.4 ± 11.1 g, LibAirty with HS at 16.4 ± 7 g, and LibAirty standalone at 11.3 ± 4.1 g. All 3 sessions with LibAirty were not statistically significant from CPT. All sessions had statistically significant higher mucus amounts compared with HFCWO (Wilcoxon signed rank test, *p* < 0.01).

No adverse events were recorded during the study. The sputum collected from each participant during each treatment is presented in Figure 1.

Lung function values for phase one pre- and post-treatment were similar without statistically significant differences.

Treatment-induced fatigue had an average score of 3/10 following all three treatments; moreover, dyspnea scores post-treatment were similar for all three treatments (3/10).

## 4. Discussion

Our study is the first study to investigate the use of the LibAirty Airway Clearance System, a novel airway clearance system, i.e., compared to an HFCWO device and our center’s common practice care (respiratory PT) for PwBE.

This study demonstrates that within a single airway clearance therapy session, the LibAirty system was equal to the common practice physiotherapy of our center and superior to the HFCWO for the main outcome of mucus expectorated in 20 min treatments.

A previous published review [30] investigated the endpoints of 70 studies on airway clearance for PwCF and found the average amount of mucus expectorated during airway clearance sessions to be 12.43 g for wet sputum. Furthermore, when split into PEP treatments into sub-groups, help expectorate the most sputum with an average of 13.76 g, while coughing alone was found to expectorate an average amount of 9.82 g. Those results correspond with the results of our study, where we found slightly higher amounts of mucus expectorated by the LibAirty system, with the BiPAP and the PT treatment producing an average amount of 14.35 g and 15.9 g, respectively. The HFCWO treatment produced the lowest yield with 4.45 ± 3.28 g expectorated. Furthermore, during the second phase of the study, both arms showed similar results to our PT session, with an average amount of 11.3 ± 4.1 g for the standalone and 16.4 ± 7 g for the LibAirty with HS 3%. The result from our PT arm of the study is in line with the results from Holland et al. [31], who reported an average of 15.3 g sputum expectorated during a 30 min airway clearance treatment using a BiPAP device.

To the best of our knowledge, the phase two arm of the LibAirty airway clearance system was the first to document the mucus volume of an airway clearance session with HS 3%. Anuradha et al. [32] studied the effectiveness of HS 3% on 10 children with non-CF bronchiectasis and found a reduction in exacerbation, and higher lung functions (FEV1, FVC) after 8 weeks [32]. In our study, the LibAirty system was found to be non-inferior to the PT arm of our study, and higher than the average yield of ACTs when compared to Chapman et al.’s review [30].

Our results of sputum expectorated using the HFCWO showed smaller amounts of expectorated mucus compared to coughing alone. The results are in concordance with a study by Leemans et al. [33], who compared two types of HFCWO devices in a 30 min treatment, finding similar amounts of expectorated mucus for the two HFCWO devices at 6.53 g and 5.8 g.

We believe the advantage in the amount of expectorated mucus obtained by the LibAirty system is due to the system’s ability to guide the patient’s breathing pattern. Similarly to AD, LibAirty promotes breathing at different lung volumes, creating shearing forces that lead to improved mucus clearance [16].

When observing the vicious cycle of respiratory therapist professionals, high demand leading to increased burnout causes higher dropout rates and an increase in load on the rest of the professionals available. The healthcare system must look for different directions to close the increasing gap and to break the cycle. Easy-to-implement non-inferior technological solutions promoting self-administered ACT are an important part of the solution.

### Study Limitations

The main limitation of this study is the fact that it is a single-center study with one clear method of respiratory physiotherapy treatment. In addition to that, twenty patients were enrolled in the study, which might limit the exploration of heterogeneity. Furthermore, the subjects were familiar with the different airway clearance modalities in this study and were not blinded to the treatment, which could lead to bias by the subjects.

## 5. Conclusions

Our study demonstrates that the LibAirty airway clearance system is superior in expectorating mucus compared to HFCWO. Additionally, its performance is comparable to that of treatments provided by experienced pulmonary physiotherapists, positioning it as a promising self-administered tool for airway clearance management. The LibAirty system is a practical and effective solution for individuals with cystic fibrosis (CF) and non-bronchiectasis conditions alike, suitable for use as a standalone therapy or in combination with other techniques or devices.

Innovations like the LibAirty system are vital in addressing the evolving challenges of modern healthcare. As the prevalence of chronic respiratory conditions continues to grow, there is an increasing need for effective, accessible, and user-friendly airway clearance solutions. The aging population and the decline in the availability of specialized caregivers further amplify the importance of such advancements. Medical devices like the LibAirty system not only improve the quality of life for patients but also contribute to reducing the strain on healthcare systems by offering reliable, at-home therapeutic options.

Future research should focus on evaluating the long-term clinical outcomes and broader applications of the LibAirty system. Such studies will help solidify its role as an integral component of a sustainable healthcare ecosystem that prioritizes innovation and patient-centered care.

## Figures and Tables

**Table 1:** Subject baseline characteristics.

Subject Number	Diagnosis	Gender	Height (cm)	Weight (kg)	BMI (kg/m^2^)	Oxygen Saturation	Fev1 (% of Predicted)	FVC (% of Predicted)
**1**	CF	M	160	62	24	98	99	9800%
**2**	CF	M	172	63	21	98	51	78
**3**	CF	F	156	46	19	99	57	76
**4**	CF	M	167	63	23	96	85	109
**5**	non CF	M	175	95	31	98	92	98
**6**	non CF	M	180	85	26	96	78	102
**7**	CF	F	170	60.6	21	96	28	59
**8**	non CF	M	182	86	26	93	57	77
**9**	CF	F	164	61	23	97	58	79
**10**	CF	M	171	62.4	21	96	62	74
**11**	non CF	F	153	46.5	20	90	36	39
**12**	non CF	F	163	57	21	98	73	85
**13**	CF	F	162	43	16	94	36	47
**14**	CF	F	164	50	19	93	55	70
**15**	non CF	F	156	70	29	96	97	109
**16**	non CF	F	161	66	25	96	81	101
**17**	non CF	F	164	52	19	98	75	101
**19**	non CF	M	180	78	24	97	62	94
**20**	non CF	F	158	49.6	20	94	56	71
**21**	non CF	F	154	44	19	98	118	115

## Data Availability

Data is contained within the article.
